# Factors influencing decision-making for caesarean section in Sweden – a qualitative study

**DOI:** 10.1186/s12884-018-2007-7

**Published:** 2018-09-17

**Authors:** Sunita Panda, Deirdre Daly, Cecily Begley, Annika Karlström, Birgitta Larsson, Lena Bäck, Ingegerd Hildingsson

**Affiliations:** 1 School of Nursing and Midwifery, Trinity College Dublin, 2 Clare Street, D02 CK80 Dublin, Ireland; 2 School of Nursing and Midwifery, Trinity College Dublin, 24 D’Olier Street, Dublin, D02 T283 Ireland; 3 Sahlgrenska Academy, University of Gothenburg, Gothenburg, Sweden; 4 Department of Nursing, Mid Sweden University, 86170 Sundsvall, Sweden; 5 Department of Women’s and Children’s health, Uppsala University, Uppsala, Sweden

**Keywords:** Caesarean section, Decision-making, Midwives, Obstetricians, Normal birth, Nulliparous, Qualitative, Midwife-led care

## Abstract

**Background:**

Rising rates of caesarean section (CS) are a concern in many countries, yet Sweden has managed to maintain low CS rates. Exploring the multifactorial and complex reasons behind the rising trend in CS has become an important goal for health professionals. The aim of the study was to explore Swedish obstetricians’ and midwives’ perceptions of the factors influencing decision-making for CS in nulliparous women in Sweden.

**Methods:**

A qualitative design was chosen to gain in-depth understanding of the factors influencing the decision-making process for CS. Purposive sampling was used to select the participants. Four audio-recorded focus group interviews (FGIs), using an interview guide with open ended questions, were conducted with eleven midwives and five obstetricians from two selected Swedish maternity hospitals after obtaining written consent from each participant. Data were managed using NVivo^©^ and thematically analysed. Ethical approval was granted by Trinity College Dublin.

**Results:**

The thematic analysis resulted in three main themes; ‘Belief in normal birth – a cultural perspective’; ‘Clarity and consistency – a system perspective’ and ‘Obstetrician makes the final decision, but...’, and each theme contained a number of subthemes. However, ‘Belief in normal birth’ emerged as the core central theme, overarching the other two themes.

**Conclusion:**

Findings suggest that believing that normal birth offers women and babies the best possible outcome contributes to having and maintaining a low CS rate. Both midwives and obstetricians agreed that having a shared belief (in normal birth), a common goal (of achieving normal birth) and providing mainly midwife-led care within a ‘team approach’ helped them achieve their goal and keep their CS rate low.

## Background

There are global concerns about the rising rate of caesarean section (CS) with wide variation in rates across countries [1] and no evidence of associated reductions in morbidities or mortalities [2]. Analysis of trends in CS rates from 121 countries indicates that rates increased from 6.7% in 1990 to 19.1% in 2014, representing an absolute increase of 12.4%. There is an increase in CS rates in developed countries (40% increase from 1993 to 2003, and an approximate 11% increase from 2003 to 2013), with the rates of CS remaining constantly higher than the World Health Organization’s (WHO) recommendations [3]. A comparison of trends in rates between developed and underdeveloped countries shows that the increase was 14.6% (from 6.3% in 1990 to 20.9% in 2014) in underdeveloped, and 12.7% in developed countries (from 14.5% in 1990 to 27.2% in 2014) [1]. The concern surrounding this striking increase in CS rates is due to the documented morbidity caused by CS [4], including increased readmission rates [5] with their concomitant costs [6].

Analysis of European data indicated that CS rates ranged from 14.8% in Iceland to 52.2% in Cyprus [7]. Despite the rising trend in rates in many countries, Sweden’s CS rate of 17% in 2010 remains among the lowest in Europe [8]. Birth choice UK (2002) has defined “normal birth” as one which starts naturally and does not involve any medical or technological intervention [9]. This working definition of normal birth thus includes spontaneous onset of labour, spontaneous progress of labour and spontaneous birth, and excludes induction of labour, epidural or spinal or general anaesthetic, forceps or ventouse, caesarean section, or episiotomy. In a concept analysis, Anderson (2003) defined normal birth as birth without intervention in an environment that enables choice and empowerment for the woman [10]. Traditionally, a culture of belief in normal birth has been evident in the Swedish maternity care system where birth is viewed as a natural process [11]. Factors that influence the decision to perform a CS are often complex and poorly explained in literature [12]. Understanding these complexities is one of many steps to help stop the rise of any unnecessary CSs or prevent its over-use. Exploring the multifactorial and complex reasons behind the rising trend in CS, and the declining rates in normal birth, has become an important goal for health professionals. Asking midwives and obstetricians about their perceptions of the factors that influence the decision to perform a CS is one way of exploring the decision-making process, and conducting this research in Sweden, a country with low CS rates, offered a unique research opportunity to achieve this goal.

## Methods

### Aim

The aim of the study was to explore Swedish obstetricians’ and midwives’ perceptions of the factors influencing decision-making for caesarean section (CS) in nulliparous women in Sweden.

### Design

A qualitative design was chosen to gain in-depth understanding of the factors influencing the decision-making process for CS using focus group interviews (FGIs).

### Setting

The study was conducted in two Swedish hospitals, each with approximately 1400 to 1600 births annually and CS rates of 14%. These are typical of Swedish maternity hospitals, and the average CS rate in the country is 17% [8], much lower than European averages. Women in these two settings and in Sweden generally, book for antenatal care with midwives. When complications exist or develop, women are referred to obstetricians. Women meet the same midwife during the antenatal visits; however, they do not have a known midwife at birth as the care is fragmented between pregnancy and birth, and belongs to two different health care systems. Antenatal care is provided within the primary health care sector and intrapartum care is hospital based (secondary health care) with no continuity with the same midwife [11].

### Participants and participant recruitment

Purposive sampling, described as when researchers “intentionally select (or recruit) participants who have experienced the central phenomenon or the key concept being explored in the study” ([13] P.173), was used. So, all midwives working in the labour wards, and obstetricians involved in the decision-making process for CS, in the site hospitals, were eligible to take part. Midwifery students and obstetricians who were not involved in the decision-making process were excluded. The purposive sampling method thus selected the midwives and senior obstetricians who were directly involved in decision-making for CS on a day-to-day basis and therefore had a deep understanding of the factors influencing decision-making for CS.

A gatekeeper (research midwife) in each study site sent the study information to the clinicians (midwives and obstetricians) in the selected study sites one to two weeks before the FGIs, identified willing participants, and arranged the date, time and venues for the FGIs.

### Data collection

After obtaining written consent from each clinician four FGIs were conducted, two with midwives (site 1, *n* = 6 and site 2 *n* = 5) and two with obstetricians (site 1 *n* = 2 and site 2 *n* = 3). The interview guide included open-ended questions; questions such as *‘Tell me how CSs are defined in your hospital?’* and ‘*Tell me about your role in decision-making for CS in nulliparous women*?’ were used to open the interviews, and probing questions such as *‘Can you tell me more about that?’* or *‘Can you explain that to me in a little more detail?*’ were used to facilitate discussion. Terms such as ‘*fear of litigation*’ or ‘*skills of clinicians*’ were also used as prompts, when appropriate, to facilitate the flow of discussion. The FGIs lasted between 30 and 35 min, and were audio recorded. During the FGIs, participants discussed issues in the Swedish language occasionally, but immediately translated these into English. Notes were made on non-verbal cues immediately after each FGI [14].

The four audio recordings were transcribed by the first author. Participants’ names, when used, were removed from the transcription and a numeric participant identification number was assigned.

### Data analysis

Data from the interviews were managed and analysed using the NVivo^©^ software package. Each step of data analysis (reading and re-reading the transcripts, coding, grouping the codes and deriving themes) was performed independently by SP and DD, and the coding and categories were compared and discussed to ensure accuracy of interpretation. To ensure reliability of the findings, all the authors discussed the interpretation of the results. The step-by-step process and stages of analysis were documented in an audit trail.

## Results

**Fig. 1:**
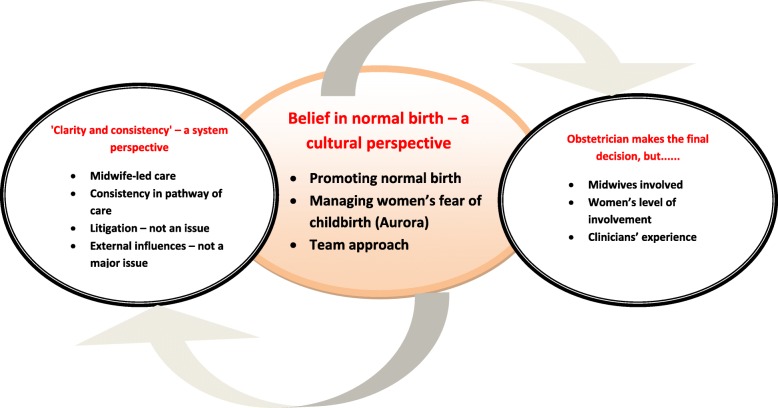
Diagram representing Swedish clinicians’ perspectives of factors influencing decision-making for CS in Sweden

The thematic analysis resulted in three main themes, each containing a number of subthemes. ‘Belief in normal birth’ emerged as the core central theme, overarching the other two themes (Fig. 1). The three themes and subthemes are described in the following section, using the clinicians’ own words to illustrate.

### Theme 1: Belief in normal birth – A cultural perspective

Participants regarded caesarean section as a procedure that should be performed only when absolutely necessary, and all agreed that this was the prevailing perspective of midwives and obstetricians in their respective hospitals, and in Sweden generally. Three subthemes were identified under this theme.

### Subtheme 1.1. Promoting normal birth

Midwives and obstetricians shared the belief that normal birth is best for women and babies, and offers the best possible outcomes. Midwives also believed that vaginal birth is associated with a more positive experience (for women) than birth by CS. CS was performed only when there was a sound justifiable reason, usually in emergency situations, and only when normal birth was no longer a safe option. Midwives agreed that obstetricians always promote normal birth, and perform CSs only when all the other measures were unsafe or have failed. Midwives also believed that avoiding induction of labour was one way to reduce unnecessary CS.

“The culture in the hospital is normal birth.” (FGI with Midwives, Site 2)

*“Women with CS have a worse birth experience than women who give birth vaginally.”* (FGI with Midwives, Site 1)

Obstetricians stated that even the general public’s opinion (in Sweden) is to promote normal birth, and midwives felt that they have a responsibility to ensure that this is explained to all pregnant women during antenatal visits.

*“I think the main factor is public opinion about what is normal and what is abnormal delivery...Most people in Sweden, I think, promote normal delivery and they think that CS is not something normal.”* (FGI Obstetricians, Site 1)

### Subtheme 1.2. Managing women’s fear of childbirth (Aurora)

Although women’s fear of childbirth, irrespective of parity, had some influence on the decision-making process, it was not considered to be a major influencing factor. Obstetricians stated that maternal request for CS was uncommon, especially in first-time mothers.

Midwives and obstetricians in both FGIs stated that Aurora, the team of midwives who counsels women with childbirth fear, played a significant role in helping women who request CS. Obstetricians confirmed that women who continue to request CS after adequate counselling are always seen by a senior obstetrician, never by junior obstetricians.

*“We have Aurora, counselling team with midwives.* [A] *woman who wish for a CS comes to this counselling programme and talks to a midwife, so we give them options like induction, pain relief, birth plan and ... a CS contract. Which means when... in labour* [if] *the woman feels that it’s too traumatic or too painful... she can by herself request for CS... And when they have this contract they feel ... in control...”* (FGI with Midwives, Site 1)

### Subtheme 1.3. Team approach

Obstetricians and midwives described a ‘team approach’ to improving outcomes in both site hospitals, and believed that group discussion and retrospective case analysis, without blaming any individual member of staff, have helped them learn from adverse outcomes and improve care.

*“Every time it goes little bit wrong, we take it on......we talk about it and analyse...and talk about how can we do better about it next time... for example,* [if] *you have an emergency CS ...we discuss afterwards...then you can learn something.”* (FGI with Midwives, Site 1)

### Theme 2 Clarity and consistency – A system perspective

Midwives and obstetricians described a consistent system of health care, with consistency in their beliefs and pathways of care, in both site hospitals. Four subthemes, which contributed to system clarity and consistency, were identified.

### Subtheme 2.1. Midwife-led care

Women always book for antenatal care with midwives, and are referred to obstetricians only when complications exist or develop, with no access for booking privately under the care of a midwife or obstetrician.

*“There is no private care here and women always go to the midwives. They book in with the midwives and the midwives refer them to the obstetricians, if and when required.”*(FGI with Midwives, Site 1)

Midwives said that midwifery staffing levels did not have an influence on the decision to perform CS; however, some obstetricians felt that the lack of experienced midwives in the labour ward did have some influence on the overall birth outcome.

*“....never had issues with short staffs. Our goal is to have one-to-one care with midwife in the room as much as we can... to avoid...*[any] *kind of distress with the woman or the couple to avoid a CS.”* (FGI with Midwives, Site 1)

*“Well, staffing might be a problem sometimes...you would like to have an experienced midwife with each woman to facilitate normal labour and maybe we can’t offer that and ... it influences the results....”* (FGI with Obstetricians, Site 1)

The midwife’s level of experience also impacted on the outcome of labour.

*“Of course... experienced midwives are the most important part of having a woman normally delivered and an experienced midwife in the room with a woman in labour ... who feels safe and confident, the woman will of course trust her...”* (FGI with Obstetricians, Site 1)

### Subtheme 2.2. Consistency in pathway of care

There was consistency in the care pathways and this approach was underpinned by clinicians’ ‘belief in normal birth’ being the best option for women and babies.

Managing early labour and avoiding induction of labour were considered vital to achieving normal birth and reducing CSs.

*“...*[for] *women to be in labour ward they must be in active labour...Because if you have any women in latent* [phase or early labour]*, there is a high risk to do something.... then a cesarean.”* (FGI with Midwives, Site 1)

Antenatal education was believed to be of vital importance, especially for women with high Body Mass Index (BMI) who were at high risk for labour and birth complications.

*“One of the reasons* [for CS in first-time mothers] *is big women. So, we educate women during pregnancy...so that when they come to delivery it’s not a big problem.”* (FGI with Midwives, Site 2)

On most occasions, women whose babies are in the breech position (at term) have an elective CS. However, if women wished to have a vaginal breech birth, clear processes are followed, and all these women undergo pelvimetry prior to deciding on the mode of birth.

*“...for breech, everyone gets a CS, unless they absolutely want to give birth* [vaginally]*. But normally they don’t. We only have few hospitals that perform breech births. You have to perform measurements,* [there] *can’t be any complications in pregnancy...”* (FGI with Midwives, Site 1)

### Subtheme 2.3. Litigation – Not an issue

Midwives and obstetricians did not have a fear of legal consequences or being blamed in cases of adverse outcomes. They believed this allows them to practise evidence-based care consistently.

*“We never had anything like that* [litigation issues] *in Sweden…The whole system is built on identifying systemic problems like education or treatment or routines...We try to see if we can educate the personnel or change routines...so that it will never happen again.”* (FGI with Obstetricians, Site 1)

*“... it’s just that kind of system, where you don’t get sued in a court. It makes it a little bit easier...”* (FGI with Midwives, Site 1)

*“...no fear of legal implications. You record everything in the journal* [women’s clinical records] *about what you are doing. Of course we have to document and do everything right but no pressure that we are going to be accused... from patients...from doctors...It is not a routine that you will think ‘I will be blamed’.”* (FGI with Midwives, Site 2)

### Subtheme 2.4. External influences – Not a major issue

While other external influencing factors sometimes exist, most participants were of the view that these had very little or no influence on clinicians’ decision-making to perform a CS.

Obstetricians in one of the study sites believed that the media can influence women’s and their families’ attitudes towards the high level of activities in hospitals and safety, but this did not impact their decision to perform CS.

*“...there might be an opinion in the society that there is a crisis at the hospital...But it does not influence our decisions about...CS.” (*FGI with Obstetrician, Site 1)

While shift times had no influence on the decision to perform CS, some midwives believed that the risk of complications increases on night shift and obstetricians believed, occasionally, that midwives’ shift changes and handover times led to some actions or procedures being delayed.

*“Change of shift is the risk. We have not many, but some examples...*[where] *decision has not been made on time because doctors were reporting to the next shift and especially…midwives’ shifts time...amniotomy gets delayed...start of oxytocin gets delayed...”* (FGI with Obstetrician, Site 1)

While availability of space in labour and birth rooms was never an issue, obstetricians in one of the study sites felt that the distance of the labour ward from the surgical theatre did, on occasions, have some influence on the timing of decision-making for CS, but not the decision to perform the CS per se.

*“Only the distance where we can perform the CS, it’s in another building, that might influence* [the timing of decision to do a CS] *sometimes...”* (FGI with Obstetrician, Site 2)

Hospital policies were not perceived as influencing clinicians’ decision-making processes; they were viewed as promoting vaginal birth.

*“The policy here is to increase...the number of normal deliveries among primipara. So it has a positive influence.”* (FGI with Obstetrician, Site 1)

### Theme 3 Obstetrician makes the final decision, but

Obstetricians were regarded as the final decision-makers for CS, but both obstetricians and midwives stated that the decision was arrived at jointly. On occasion, women’s level of involvement in the discussions, and clinicians’ experience, exerted some influence.

### Subtheme 3.1. Midwives involved

Although obstetricians make the final decision to perform a CS, midwives always played a vital role in the decision-making discussions.

*“When a woman comes to labour ward, we make a risk classification…and most of the cesareans are high risk, so* [it is] *team-work...but it’s the doctor who makes the decision.”* (FGI with Midwives, Site 1)

*“In a case where the reason for CS is...dystocia…we would discuss it with the midwife more than when it’s due to fetal distress. Even the junior colleagues may discuss it with the midwives...rather than calling…a senior consultant.”* (FGI with Obstetricians, Site 1)

### Subtheme 3.2. Women’s level of involvement

Women played very little role in decision-making when a CS was performed in an emergency. However, in non-emergency situations when it was clinically safe, it was common practice to discuss and consider women’s preferred care pathway and mode of birth.

*“Not if it is immediate* [emergency]…*but otherwise* [the woman is] *definitely involved.”* (FGI with Midwives, Site 1)

### Subtheme 3.3. Clinicians’ experience

Clinicians’ experience had an influence in the process of decision-making, and midwives felt obstetricians with limited experience tended to perform a CS sooner than more experienced colleagues.

*“...I think that some of the new doctors might not let people try for such a long time than really skilled ones.”* (FGI with Midwives, Site 2)

Consultant obstetricians stated that some senior obstetric registrars, depending on their level of experience, could make the final decision for CS, mainly in emergency situations. However, a consultant obstetrician’s availability in the labour ward, mostly during day-time, was always perceived to make a difference to the decision-making process.

*“If you are a resident* [obstetric registrar]*, and you are concerned, you will discuss with the senior. That depends on how experienced you are. If you are experienced, you make the decision by yourself.”* (FGI with Obstetricians, Site 1)

*“I think one advantage is that the consultants are always...in the labour ward... we are always available...”* (FGI with Obstetricians, Site 2)

## Discussion

A belief in normal birth, together with a multidisciplinary team approach, have been shown to have a positive impact on reducing CSs [15], including in the Swedish maternity system [11]. In this study, the main factor said to influence the mode of birth for first-time mothers was the culture of belief in normal birth.

A woman’s first birth and her overall experience is crucial to her future reproductive health and, in Sweden fear of childbirth is one of the predominant causes of women requesting for CS [16] and most of these fears are attributed to previous negative birth experiences. In the Swedish maternity system, support and counselling services are offered to women who present with fear of childbirth. No differences in CS rates are reported for women with severe fear of child birth with no counselling service, but they are reported to have increased negative birth experiences [17] and, women who receive support and counselling for fear of childbirth have greater levels of satisfaction with the care they receive [18].

The reasons why women request a CS are complex [19] and are often influenced by previous birth experience, socio-cultural factors, media and body image [19–22]. In this study, maternal request for CS because of fear of childbirth was regarded as rare among nulliparous women. While attitudes to maternal request for CS differ among midwives and obstetricians in other countries [23, 24], in the absence of any medical indication, CS on maternal request is rarely an option in Sweden [11]. Clinicians in this study were clear and consistent about counselling women who request a CS.

Fear of adverse outcomes and subsequent litigation are frequently reported factors influencing the decision to perform a CS in many OECD [25–28] and non-OECD countries [21]. Clinicians in this study, however, described litigation as having no influence on their decision-making for CS. Litigation issues and complaints in Sweden are overseen by the Medical Responsibility Board; and most issues are investigated to improve future care [11].

Despite several studies that describe a lack of cooperation and professional disagreements among midwives and obstetricians on the decision-making process for CS [21, 24, 29], findings from these FGIs show that there is ‘a team approach’ with the midwifery and obstetric teams in these two sites that helps them maintain a low CS rate.

Avoiding induction of labour was viewed as a key element in reducing CS rates. Caring for women in the latent phase of labour outside the labour ward area was seen as essential, findings similar to those of a recent Irish study [30].

Midwives are the primary pregnancy care providers in Sweden [11], and have also been shown to play a pivotal role in ensuring a positive birth experience for women [31]. Clinicians in this study viewed ‘one-to-one midwifery’ and ‘continuity of care’ as vital elements for promoting normal birth and reducing CS, like other studies [25]. Swedish maternity care is acknowledged as having a culture that believes normal birth as the optimal birth mode for women, and maternity care models that emphasise continuity of care. Continuity of care with the same known midwife has been valued by all women, especially women with fear of childbirth, through facilitating normal birth and a positive birth experience [32]. Internationally, midwife-led care has been shown to reduce CS rates [33], and costs less than obstetrician-led care [34].

Attending obstetricians privately is frequently reported as contributing to the rising CS rate [12, 21, 35]. Private organisations providing antenatal care in Sweden, with midwives as primary care providers, have emerged in recent years, mostly in urban areas but there are no private labour wards [11]. However, the clinicians in the current study indicated that there was no privatisation of maternity care in their two sites, which has helped them retain uniformity and consistency in their care for women.

As findings from FGIs can be dominated by the opinions of one, or more, group participants [36], in this study, conducting separate FGIs with midwives and obstetricians was a strength and contributed to ‘Clarity and consistency - a system perspective’ emerging as one of three core themes. All clinicians volunteered to participate and actively contributed to the discussions. The two study sites (with approximately 1400 to 1600 births annually and CS rates of 14%) are typical of maternity hospitals outside the bigger cities in Sweden. Both offer care to all women above 28 weeks of pregnancy, and have successfully maintained low CS rates. However, while the hospitals are representative of Sweden, they may not be representative of other maternity hospitals throughout Europe in general, and this is a potential limitation.

## Conclusion

Findings from the current study suggest that ‘a belief in normal birth’ offers the best possible outcome and contributes to maintaining a low rate of CS. Providing midwife-led care within a ‘team approach,’ with a common goal and shared belief in achieving normal birth, are some key elements to maintain a low CS rate. Findings from this study are highly applicable to maternity care in other countries. The implications are that, changing clinicians’ attitudes to a common and shared ‘belief in normal birth’, and acceptance of the core key issues may contribute to a reduction in CS rates and prevent the rates from rising further.

## Abbreviations

CS: Caesarean Section
FGI: Focus Group Interview
